# Supplementary figures and images for: Proteostasis modulates gene dosage evolution in antibiotic-resistant bacteria
Source: eLife. 2025 Mar 12;13:RP99785. doi: 10.7554/eLife.99785 (PMC11903035; doi:10.7554/eLife.99785)

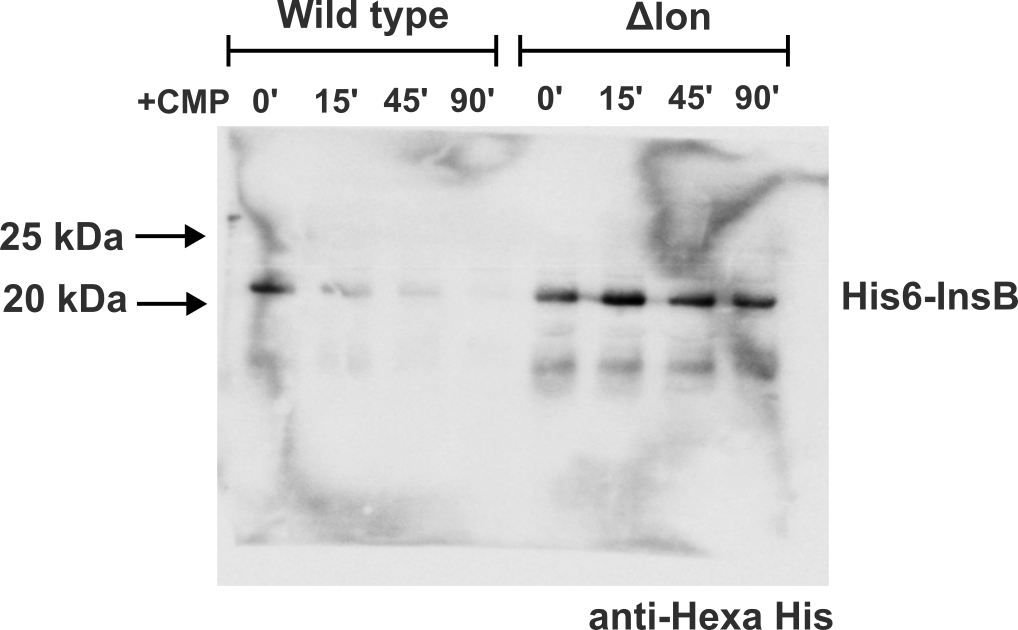

Supplement: Figure 1—figure supplement 2—source data 1. [file elife-99785-fig1-figsupp2-data1.zip › Figure 1-figure Supplement 2-InsB Annotated.tiff]

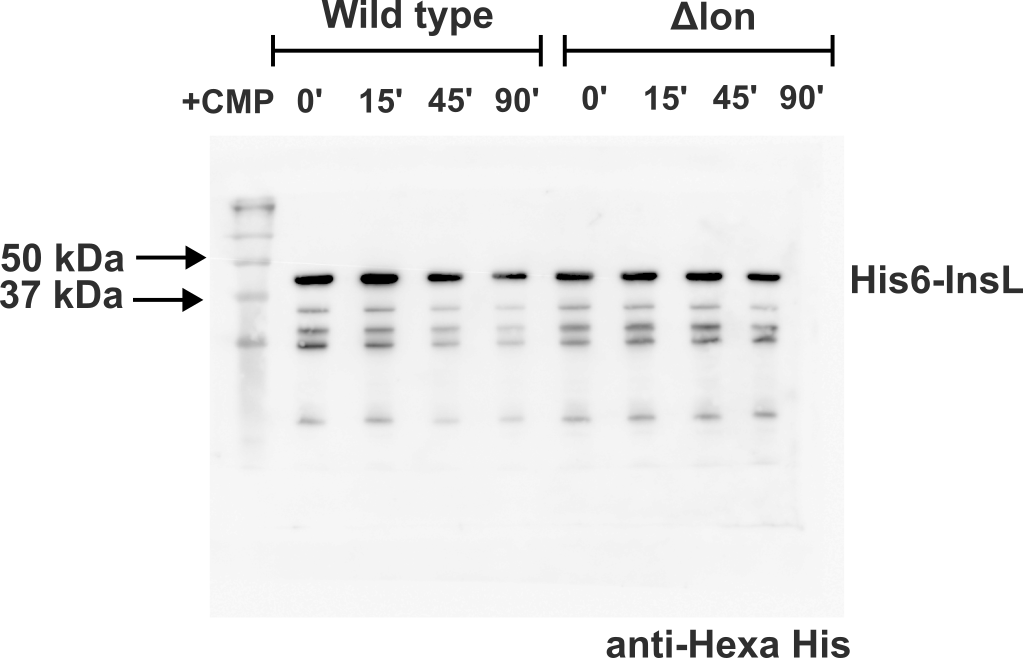

Supplement: Figure 1—figure supplement 2—source data 1. [file elife-99785-fig1-figsupp2-data1.zip › Figure 1-figure Supplement 2-InsL Annotated.tiff]

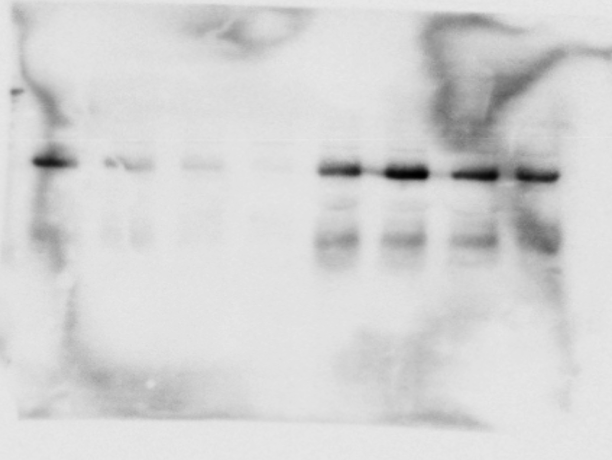

Supplement: Figure 1—figure supplement 2—source data 2. [file elife-99785-fig1-figsupp2-data2.zip › Figure 1-figure Supplement 2-InsB Raw.tiff]

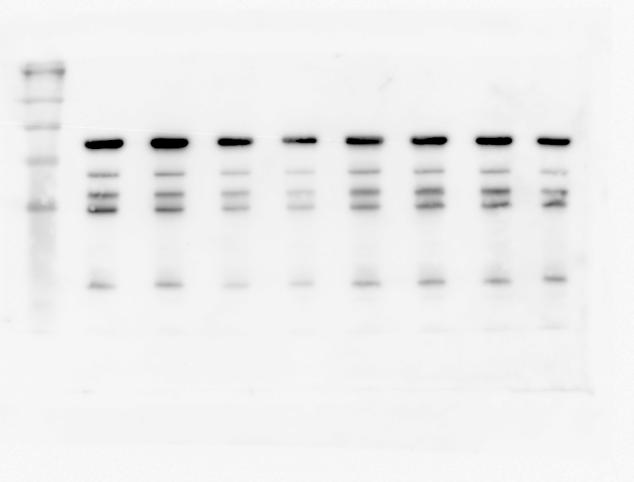

Supplement: Figure 1—figure supplement 2—source data 2. [file elife-99785-fig1-figsupp2-data2.zip › Figure 1-figure Supplement 2-InsL Raw.tiff]

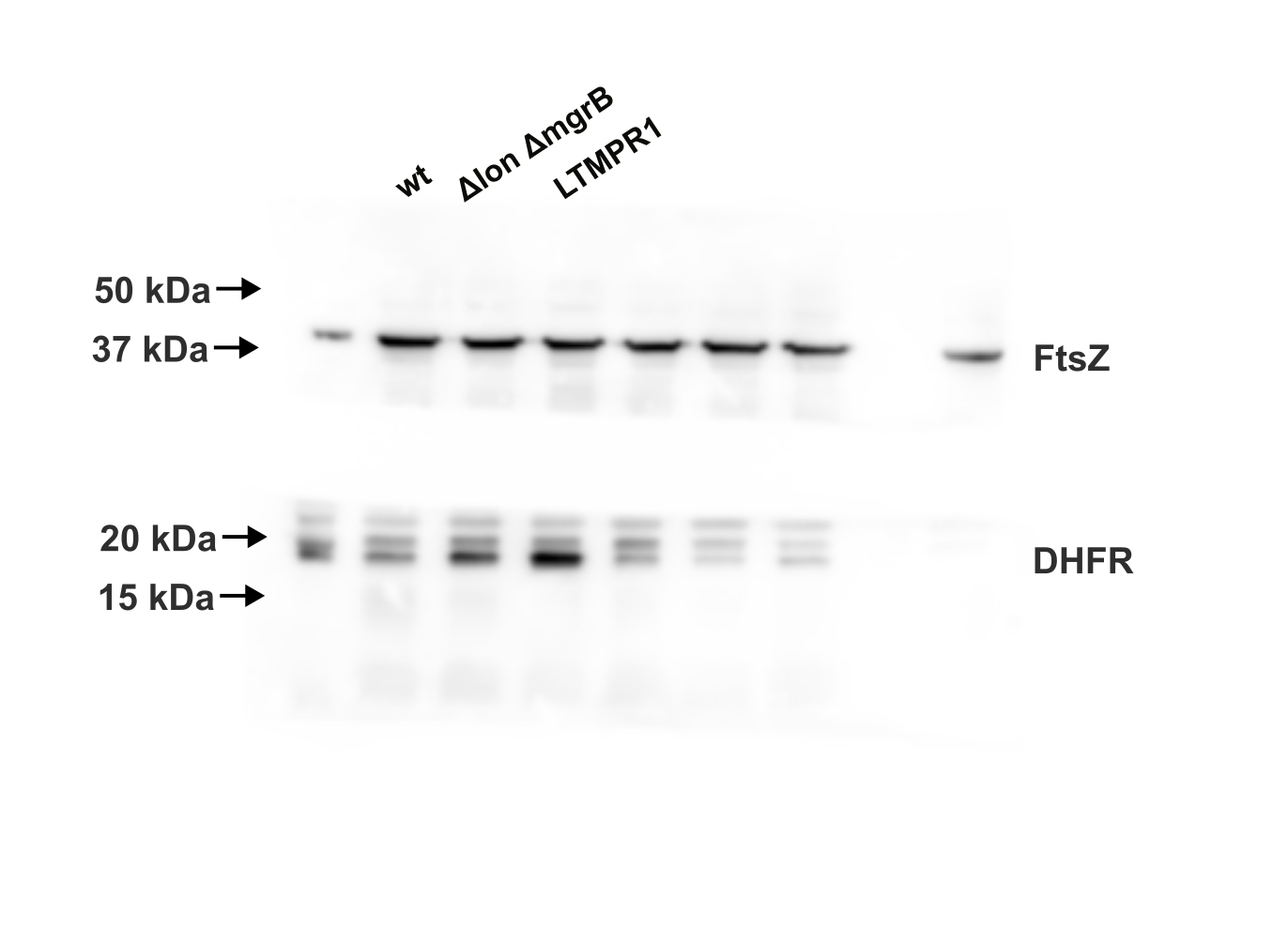

Supplement: Figure 2—source data 1. [file elife-99785-fig2-data1.zip › Figure 2-DHFR LTMPR1 Annotated.tiff]

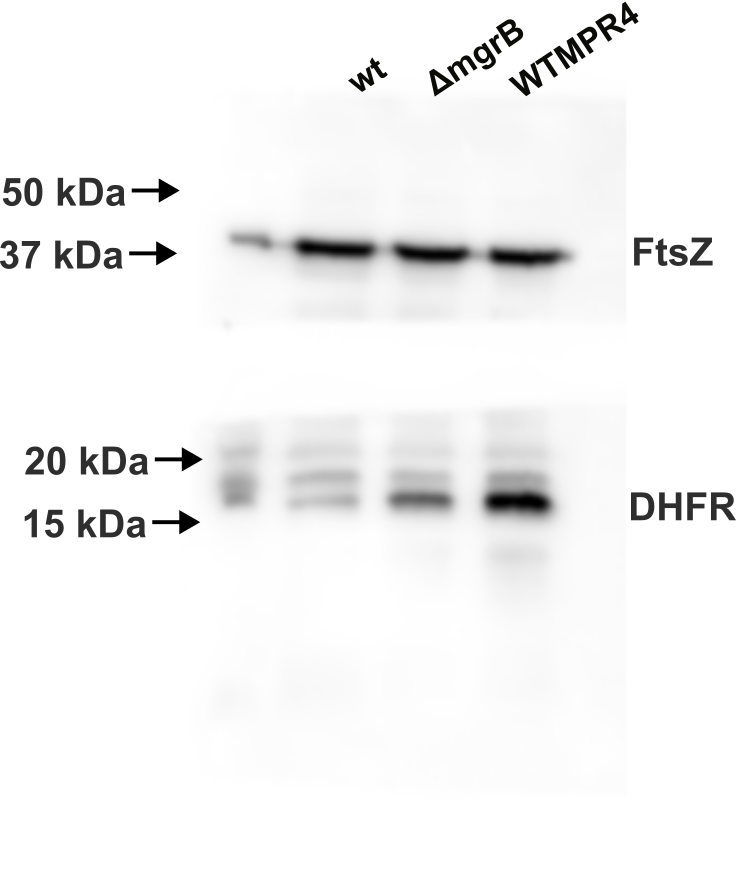

Supplement: Figure 2—source data 1. [file elife-99785-fig2-data1.zip › Figure 2-DHFR WTMPR4 Annotated.tiff]

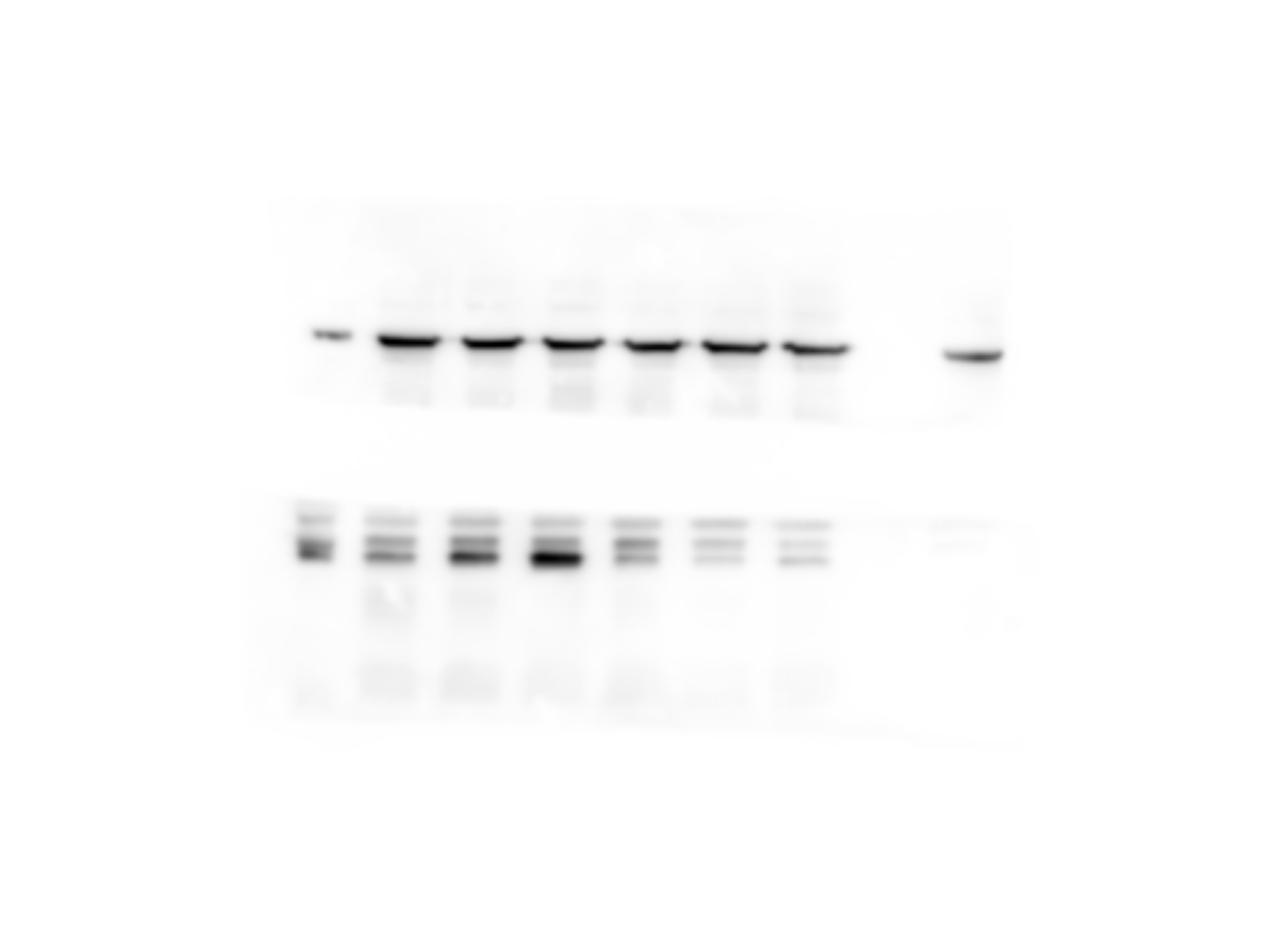

Supplement: Figure 2—source data 2. [file elife-99785-fig2-data2.zip › Figure 2-DHFR LTMPR1 Raw.tiff]

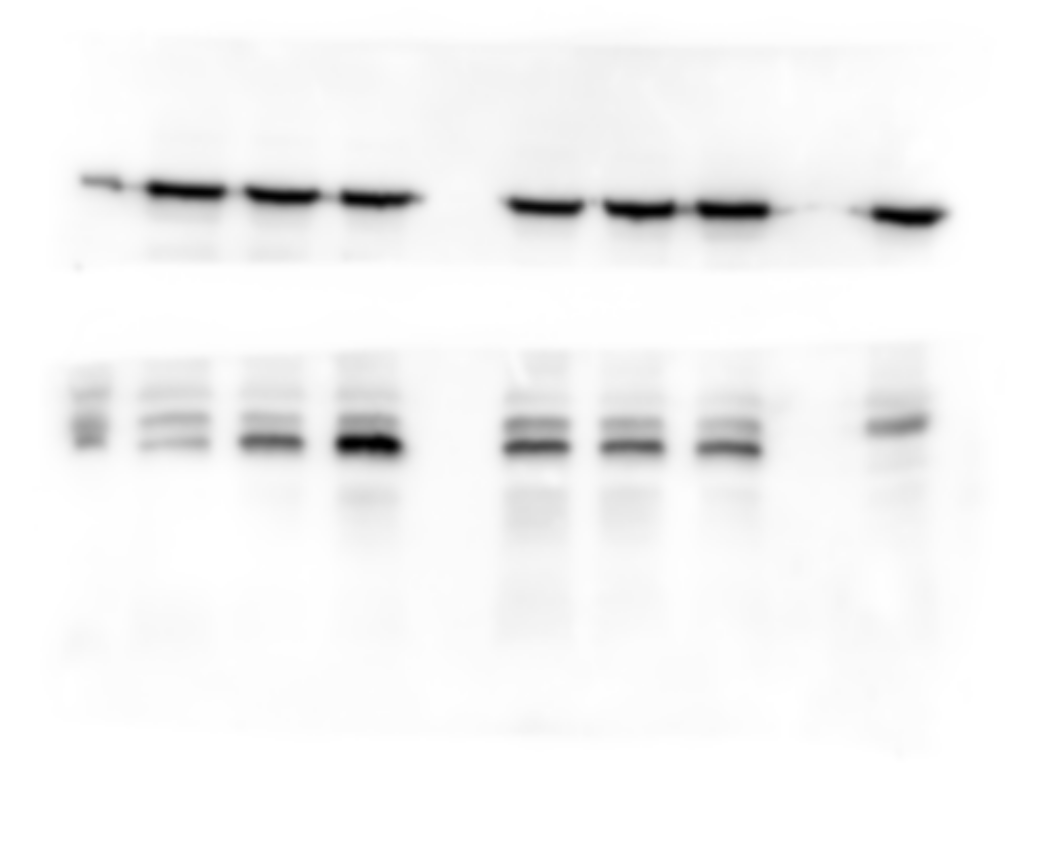

Supplement: Figure 2—source data 2. [file elife-99785-fig2-data2.zip › Figure 2-DHFR WTMPR4 Raw.tiff]

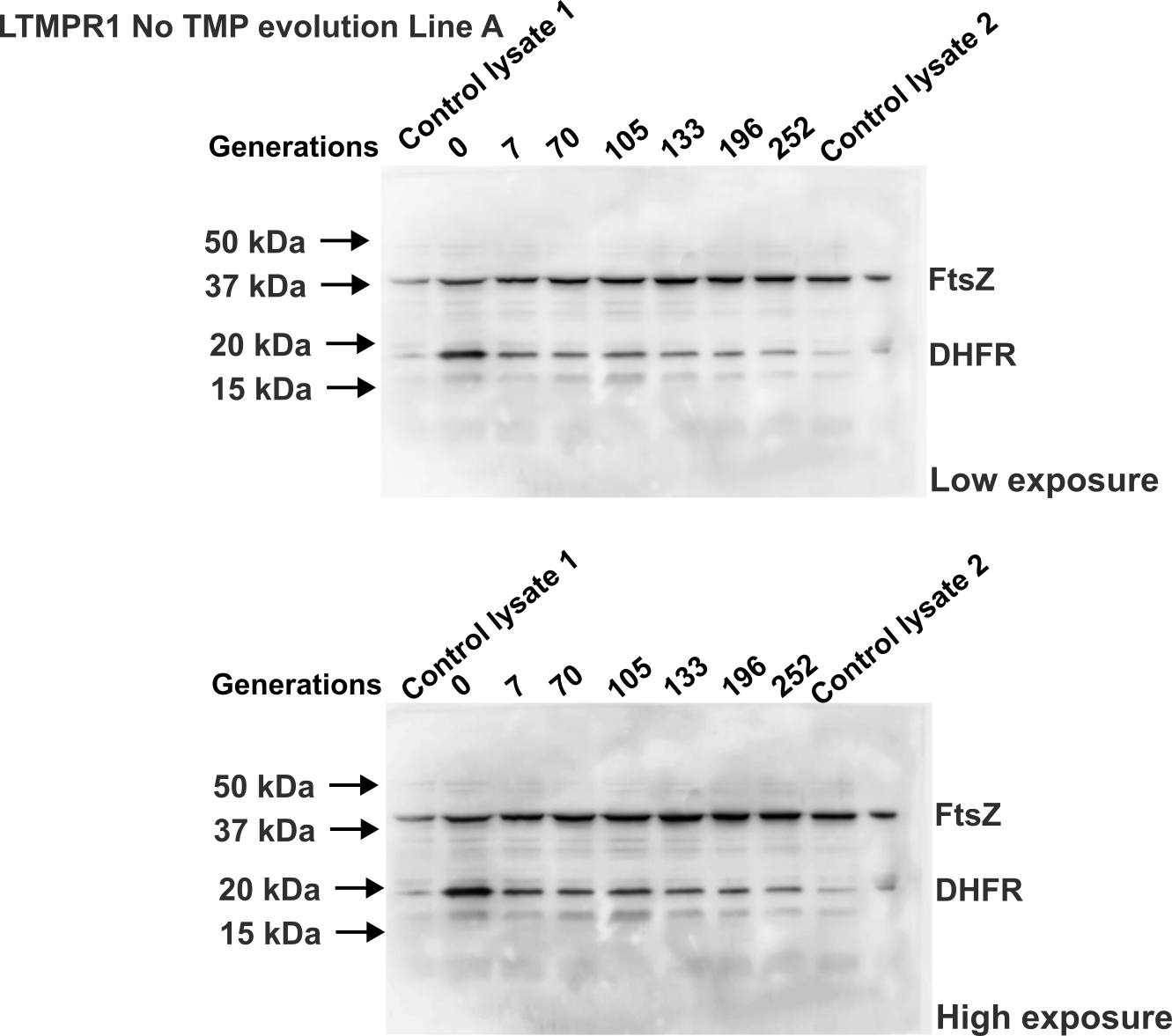

Supplement: Figure 3—source data 1. [file elife-99785-fig3-data1.zip › Figure 3-DHFR Line A Annotated.tiff]

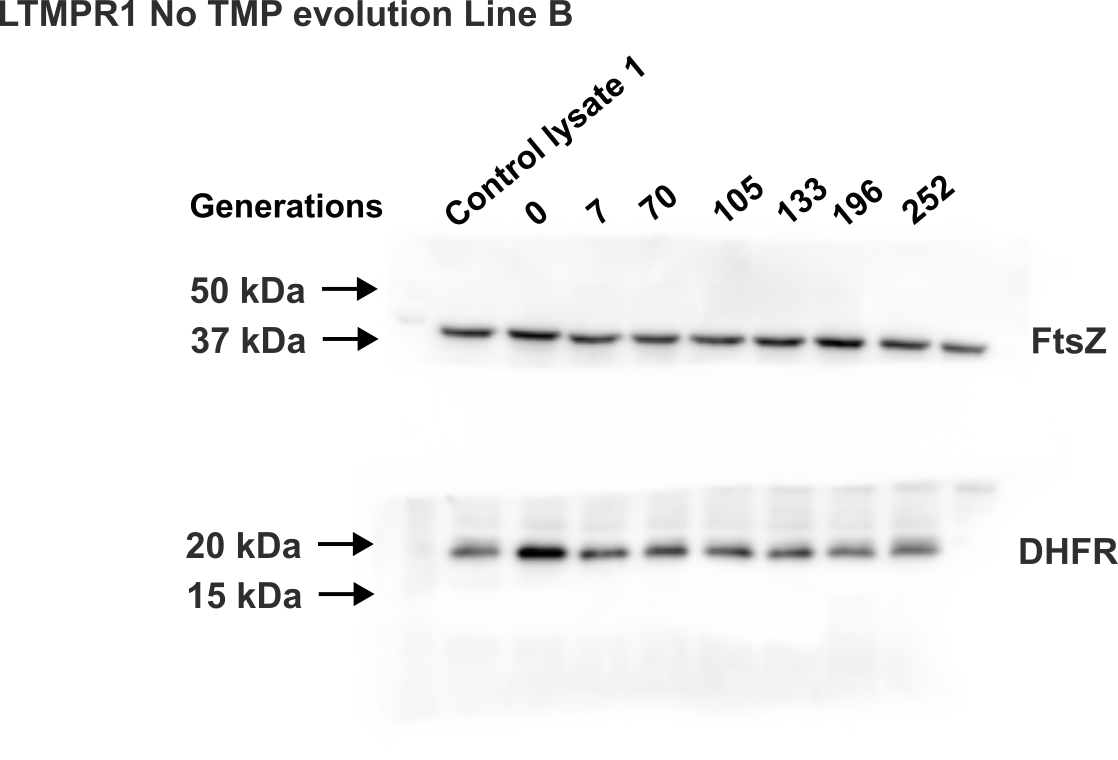

Supplement: Figure 3—source data 1. [file elife-99785-fig3-data1.zip › Figure 3-DHFR Line B Annotated.tiff]

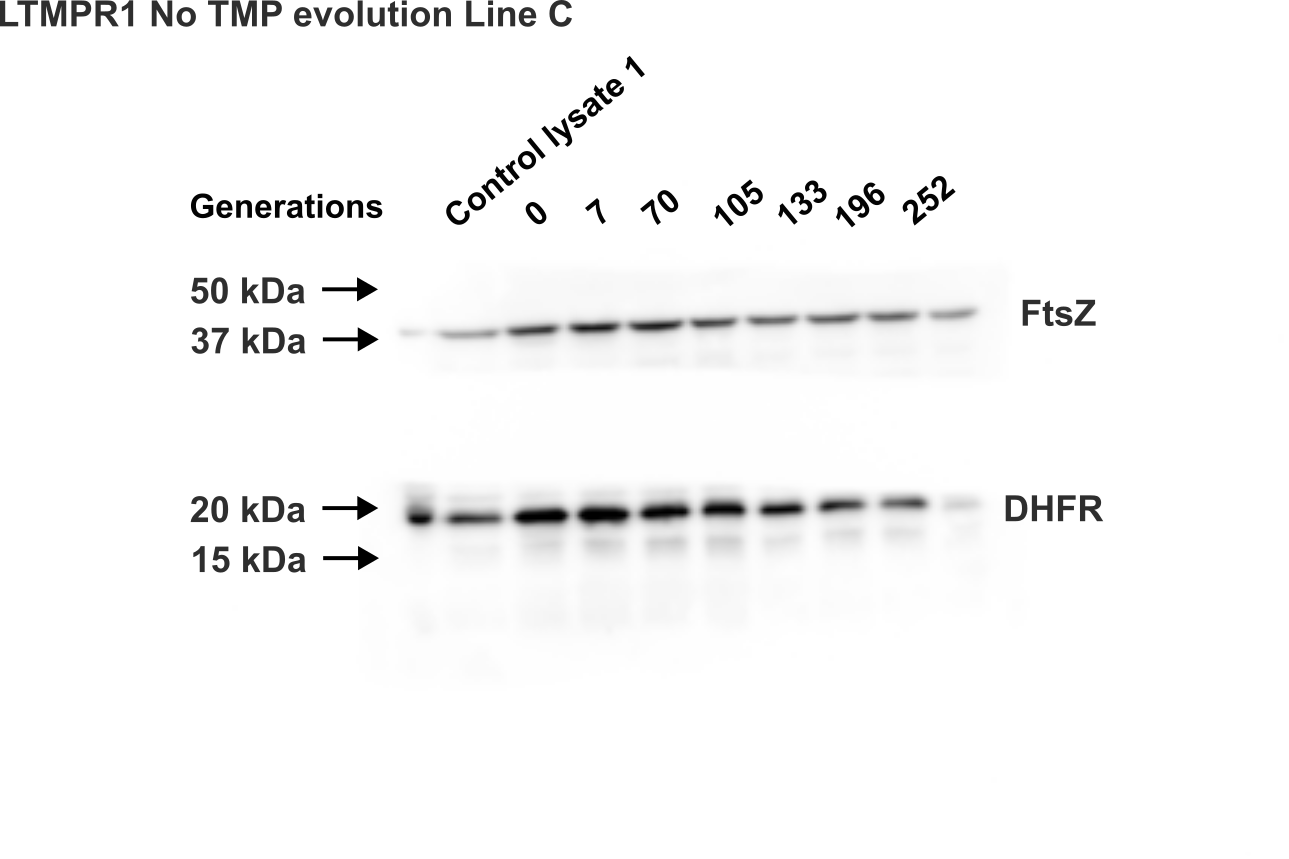

Supplement: Figure 3—source data 1. [file elife-99785-fig3-data1.zip › Figure 3-DHFR Line C Annotated.tiff]

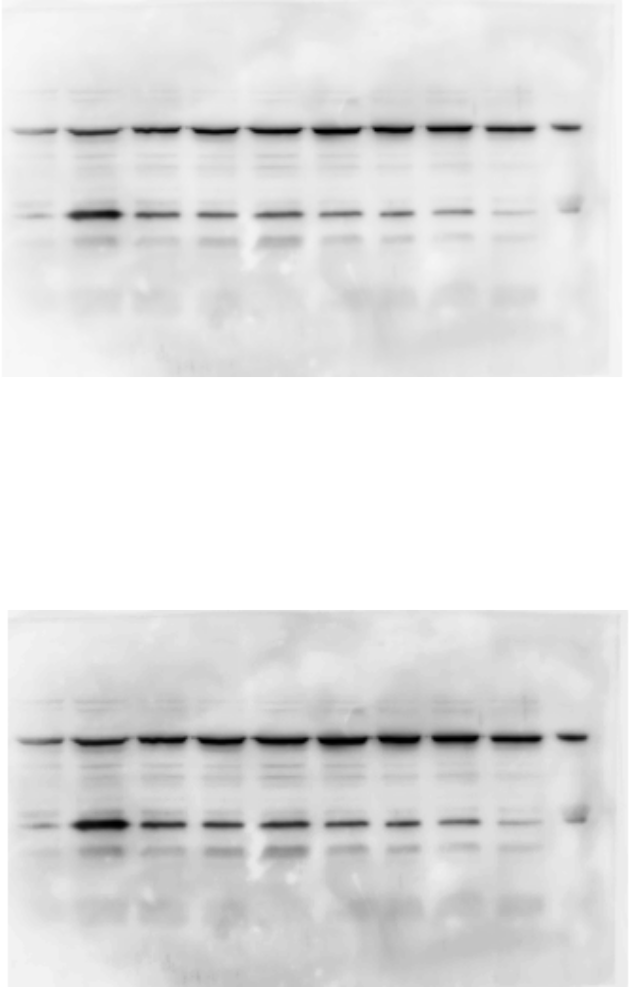

Supplement: Figure 3—source data 2. [file elife-99785-fig3-data2.zip › Figure 3-DHFR Line A Raw.tiff]

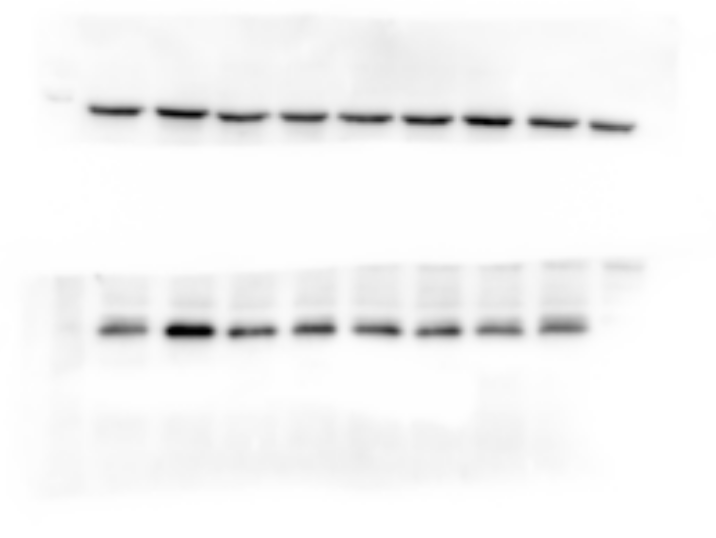

Supplement: Figure 3—source data 2. [file elife-99785-fig3-data2.zip › Figure 3-DHFR Line B Raw.tiff]

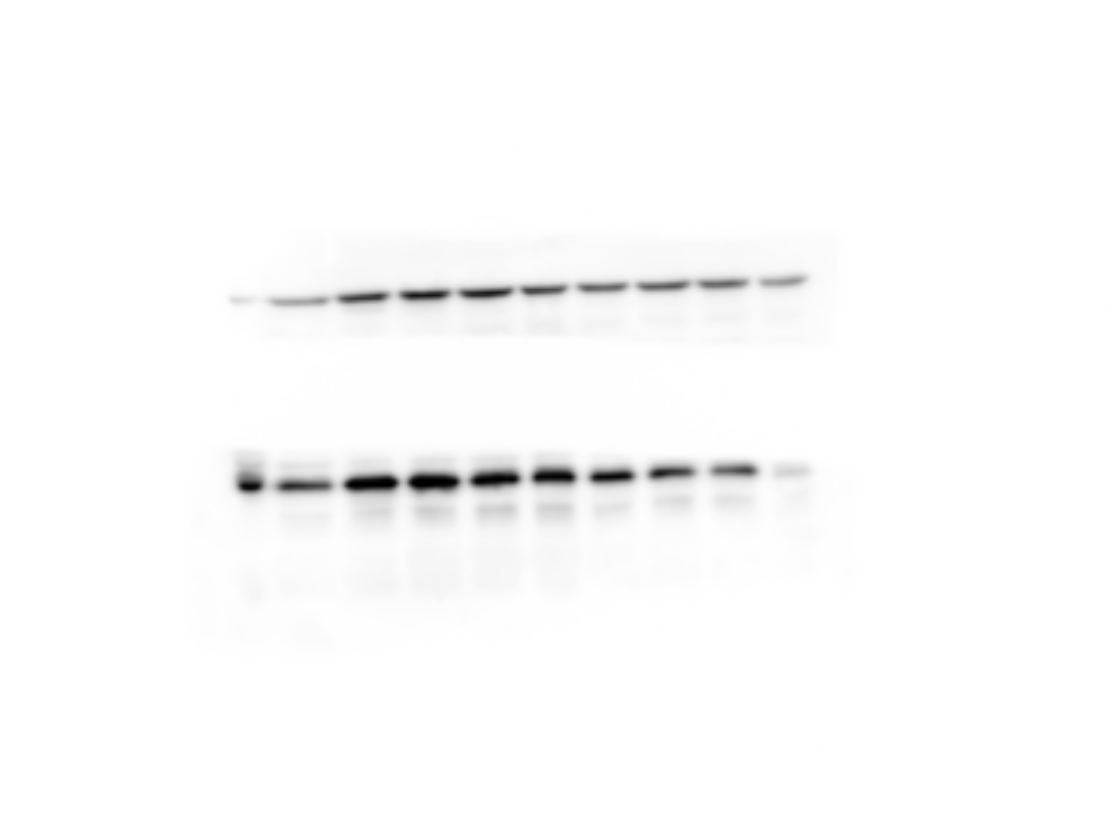

Supplement: Figure 3—source data 2. [file elife-99785-fig3-data2.zip › Figure 3-DHFR Line C Raw.tiff]

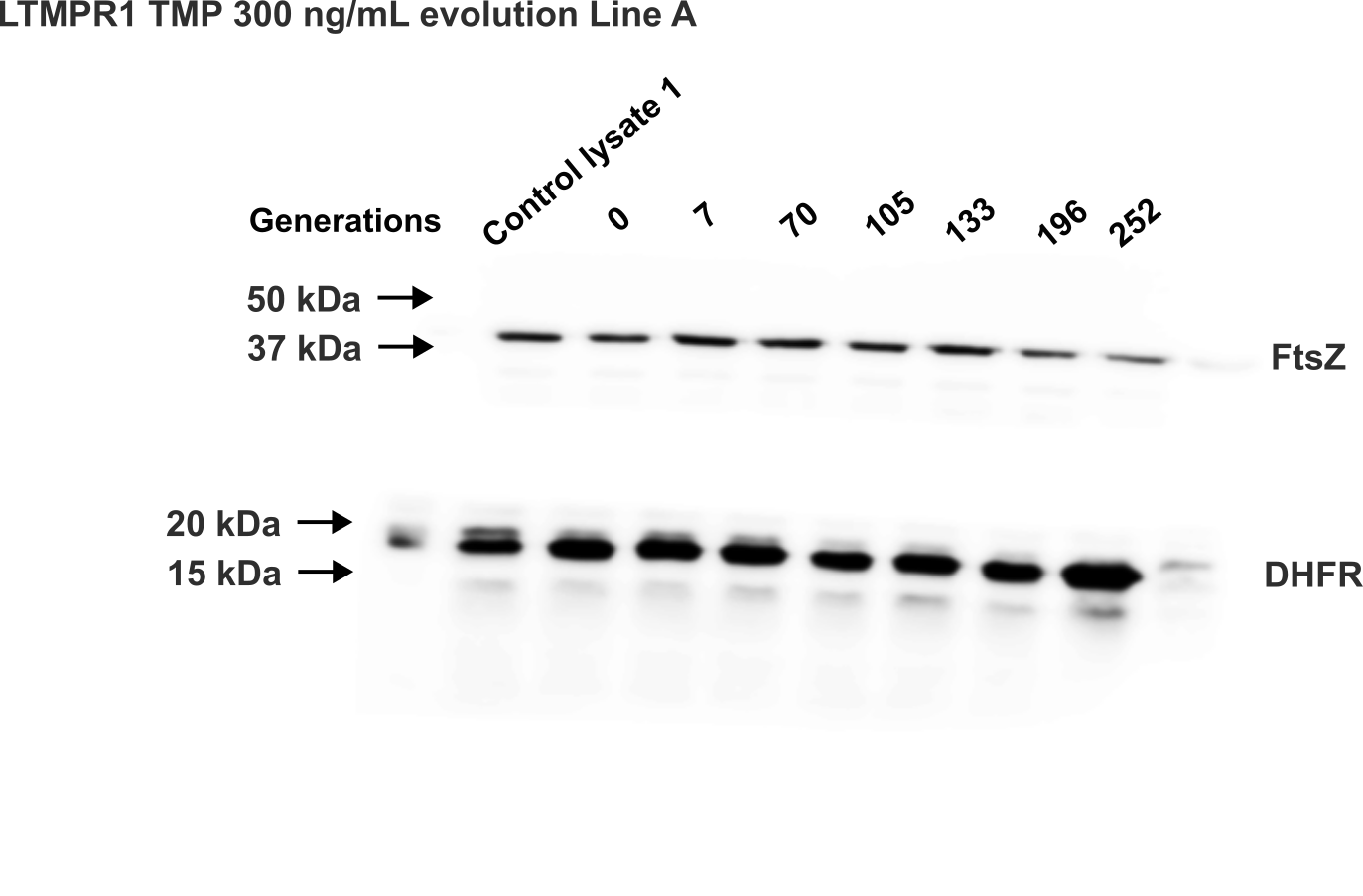

Supplement: Figure 5—source data 1. [file elife-99785-fig5-data1.zip › Figure 5-DHFR Line A Annotated.tiff]

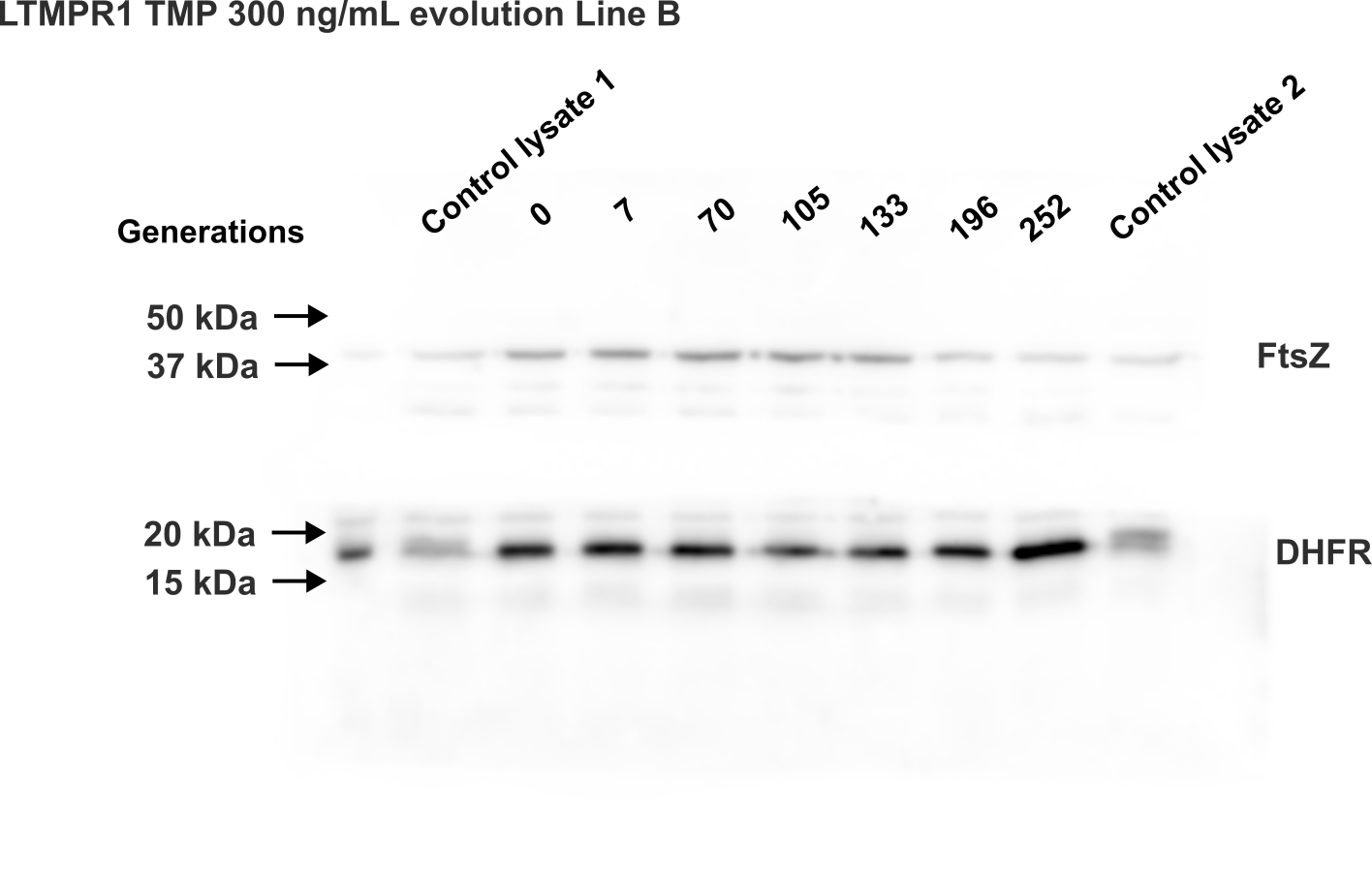

Supplement: Figure 5—source data 1. [file elife-99785-fig5-data1.zip › Figure 5-DHFR Line B Annotated.tiff]

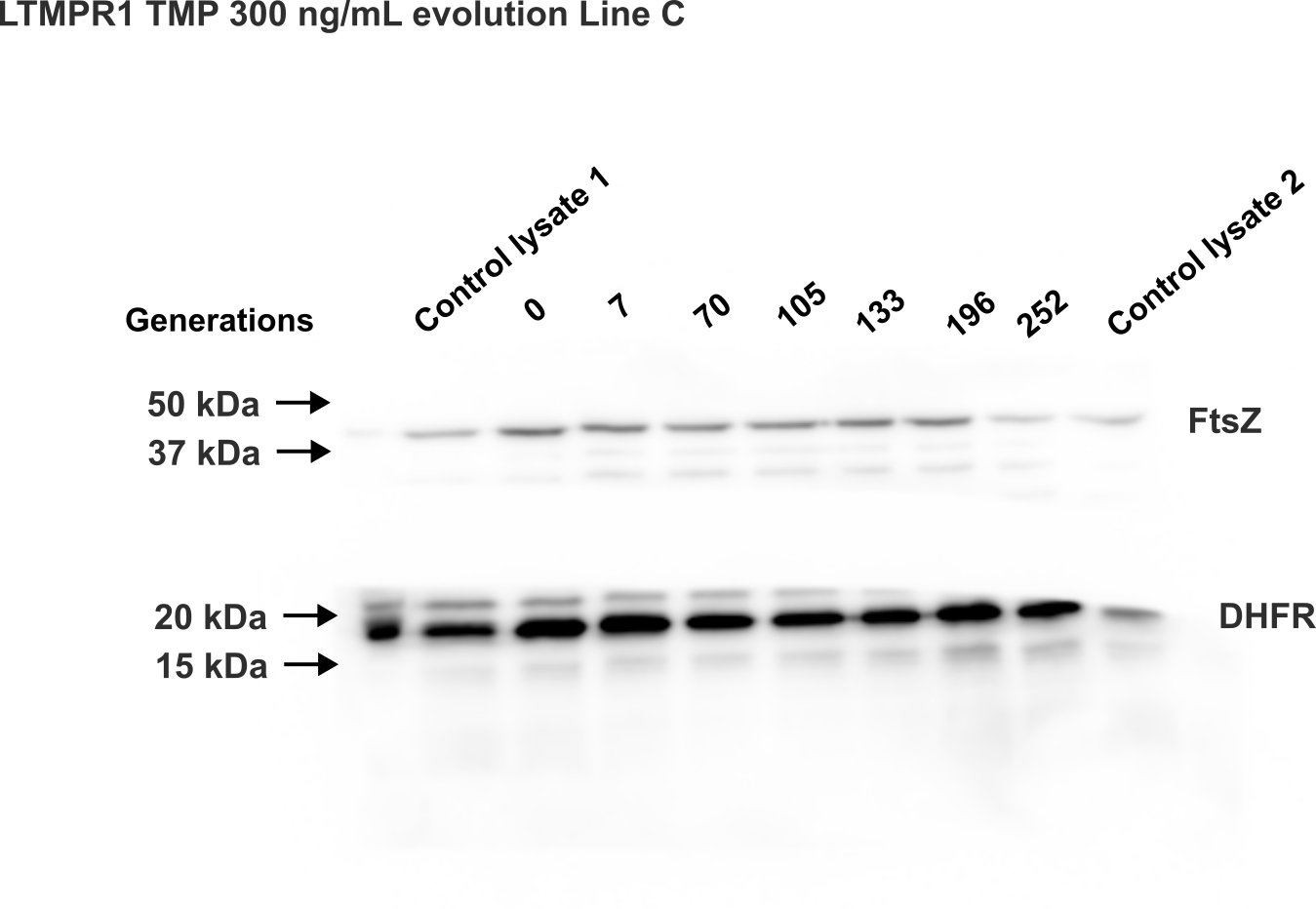

Supplement: Figure 5—source data 1. [file elife-99785-fig5-data1.zip › Figure 5-DHFR Line C Annotated.tiff]

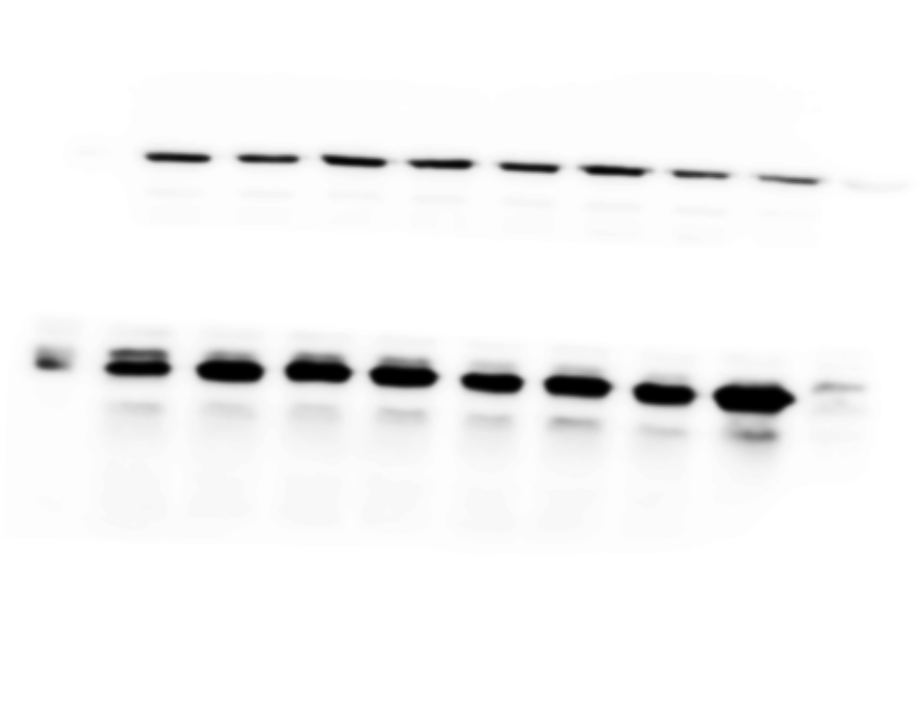

Supplement: Figure 5—source data 2. [file elife-99785-fig5-data2.zip › Figure 5-DHFR Line A Raw.tiff]

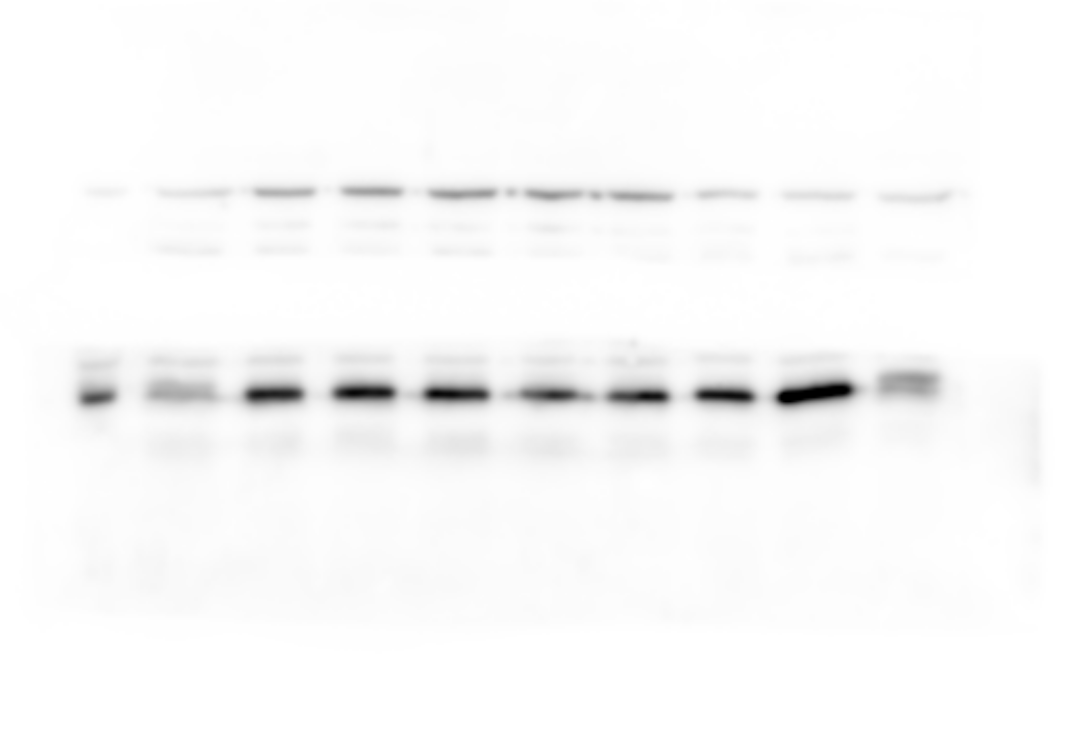

Supplement: Figure 5—source data 2. [file elife-99785-fig5-data2.zip › Figure 5-DHFR Line B Raw.tiff]

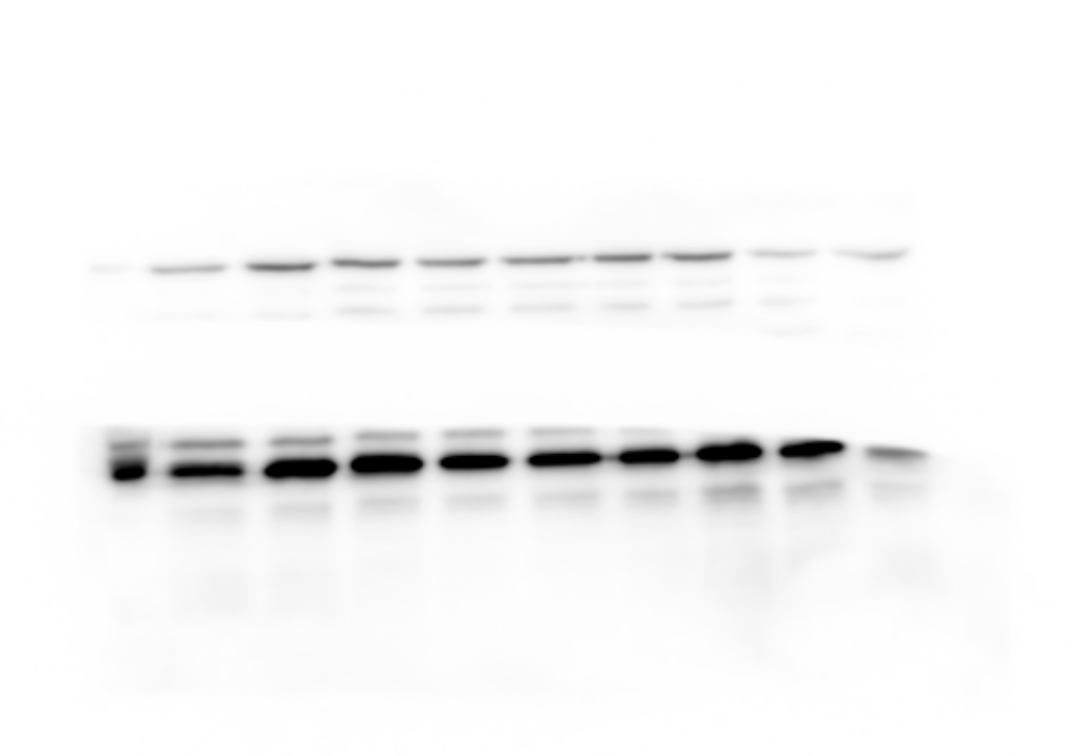

Supplement: Figure 5—source data 2. [file elife-99785-fig5-data2.zip › Figure 5-DHFR Line C Raw.tiff]

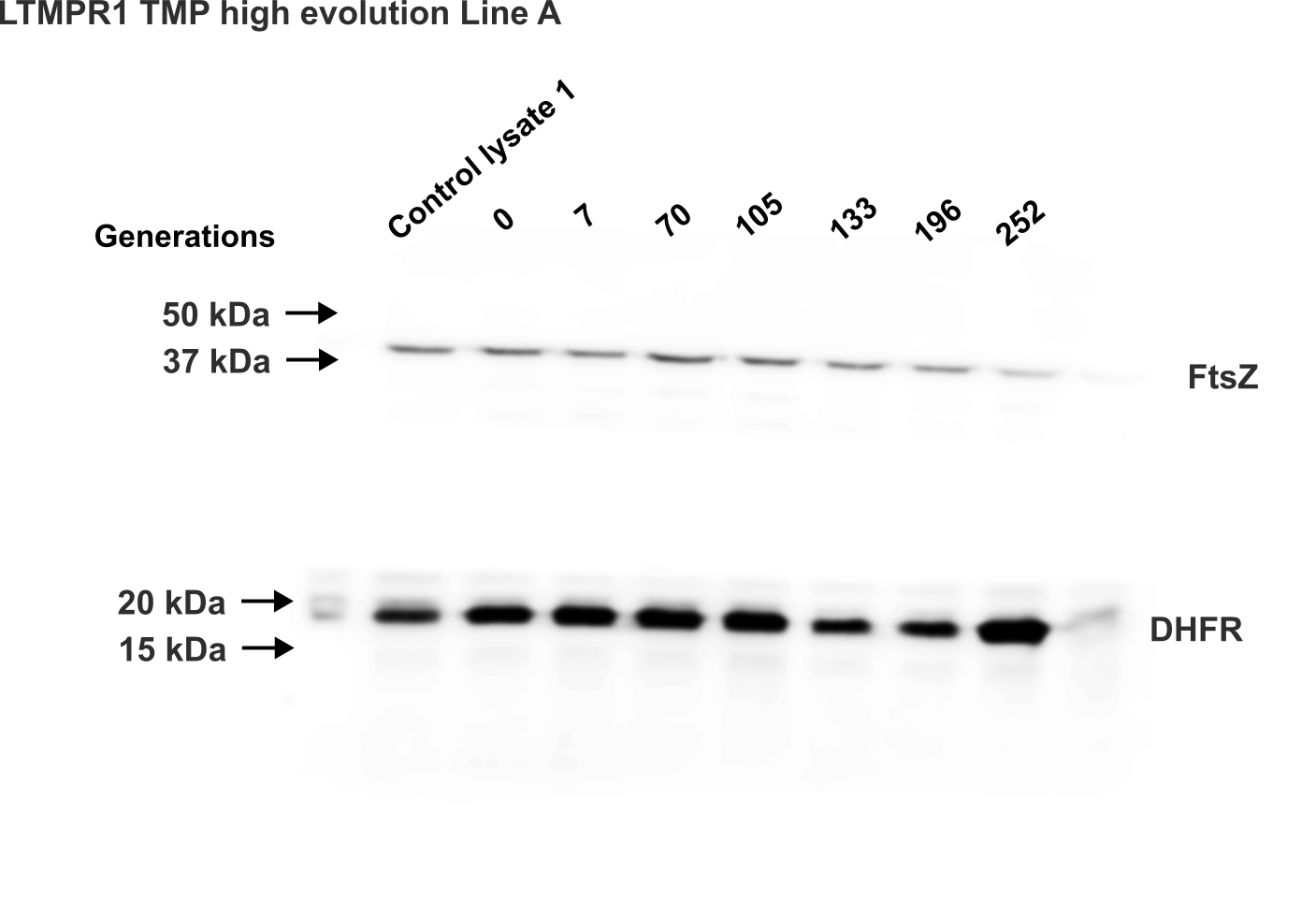

Supplement: Figure 7—source data 1. [file elife-99785-fig7-data1.zip › Figure 7-DHFR Line A Annotated.tiff]

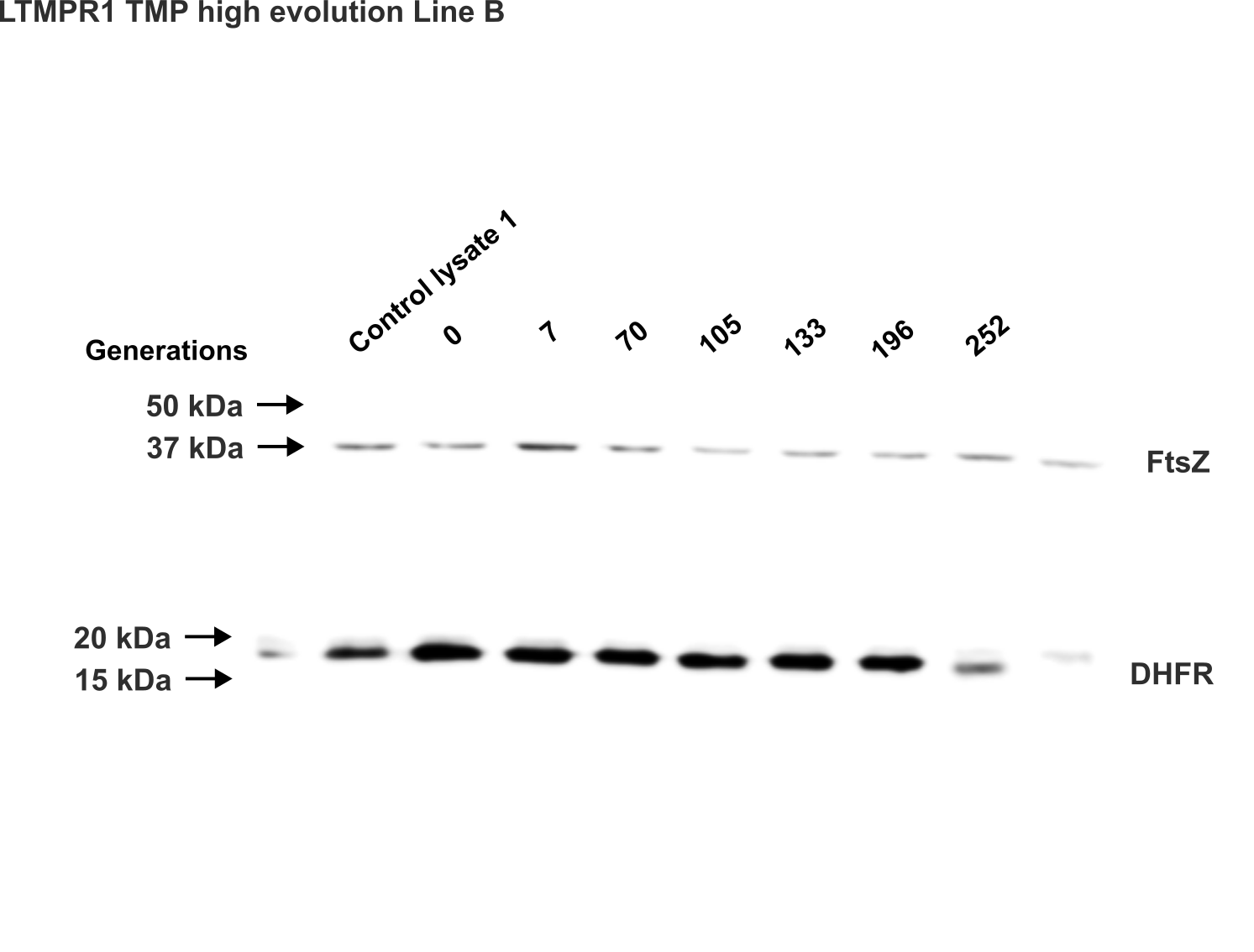

Supplement: Figure 7—source data 1. [file elife-99785-fig7-data1.zip › Figure 7-DHFR Line B Annotated.tiff]

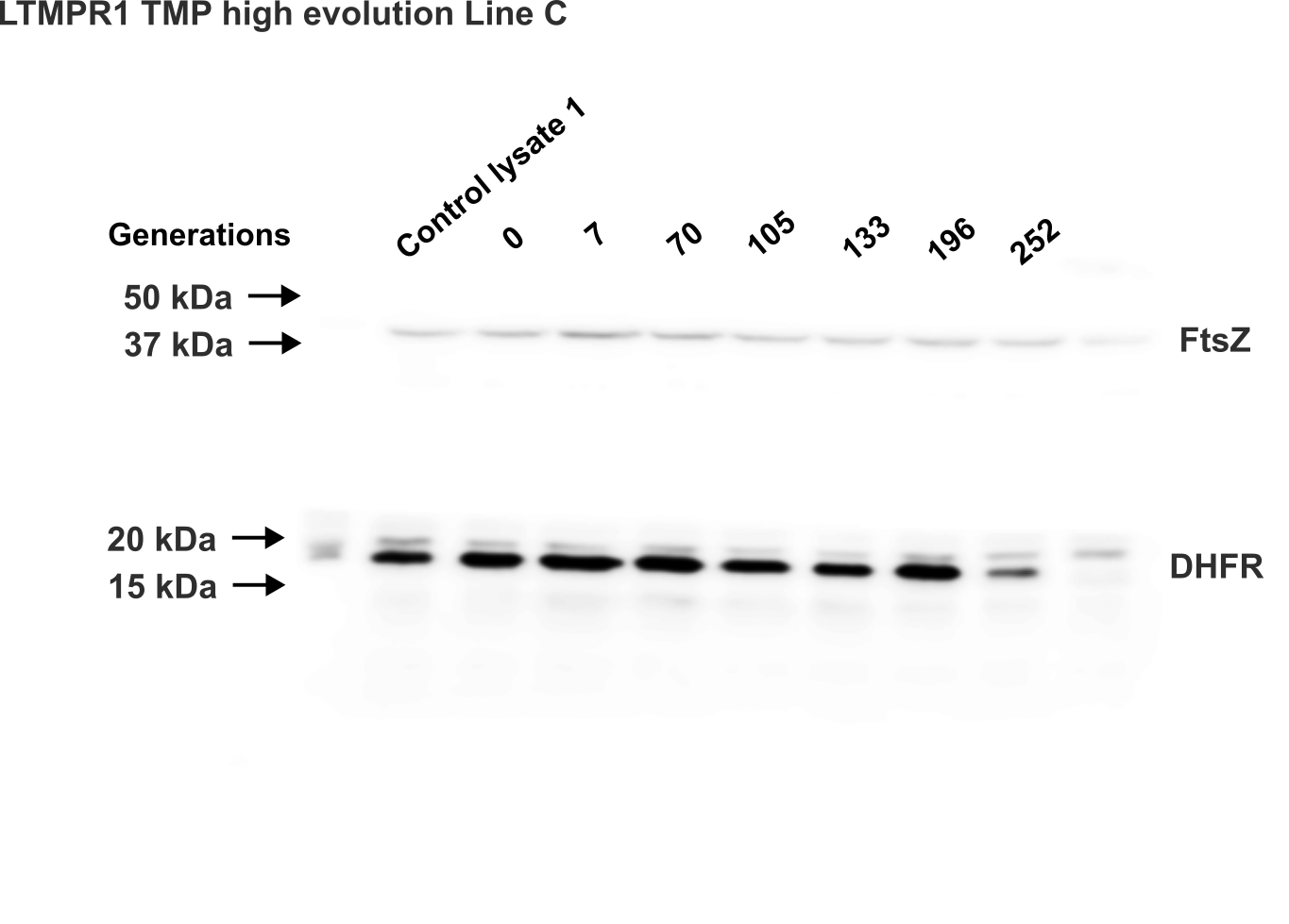

Supplement: Figure 7—source data 1. [file elife-99785-fig7-data1.zip › Figure 7-DHFR Line C Annotated.tiff]

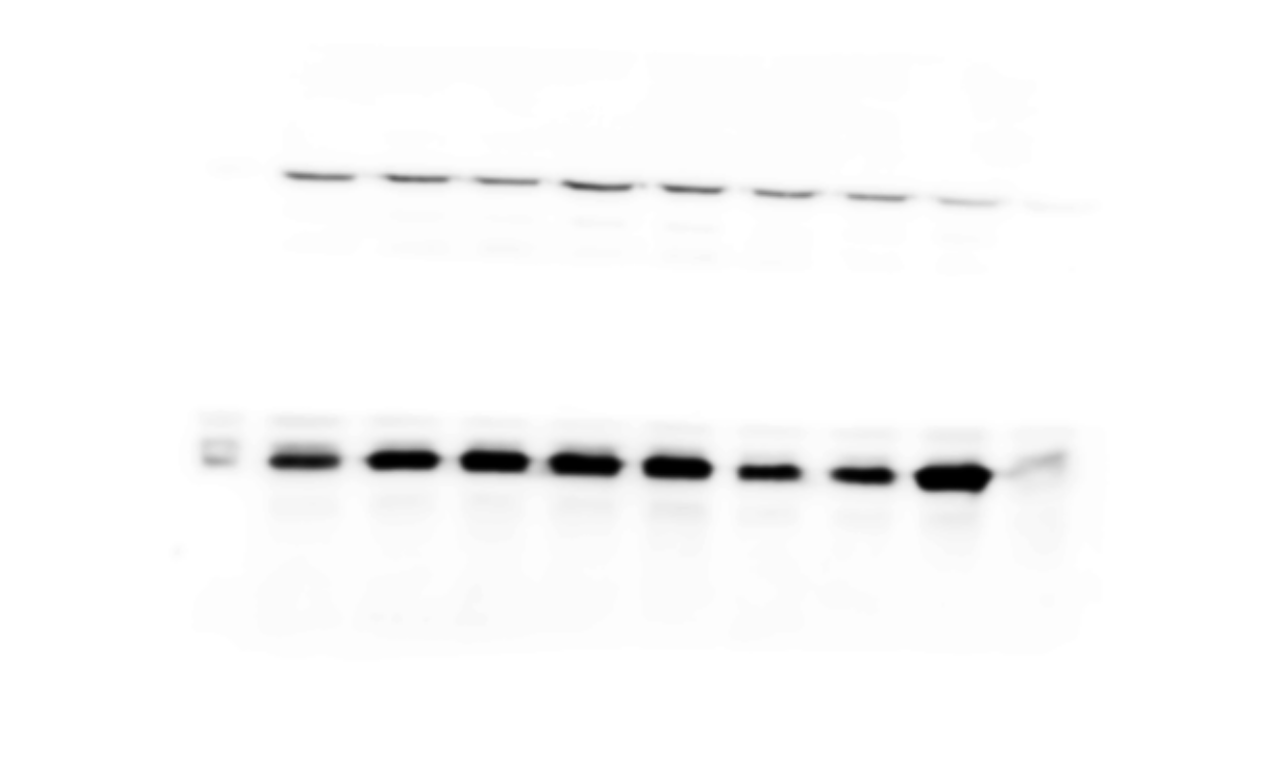

Supplement: Figure 7—source data 2. [file elife-99785-fig7-data2.zip › Figure 7-DHFR Line A Raw.tiff]

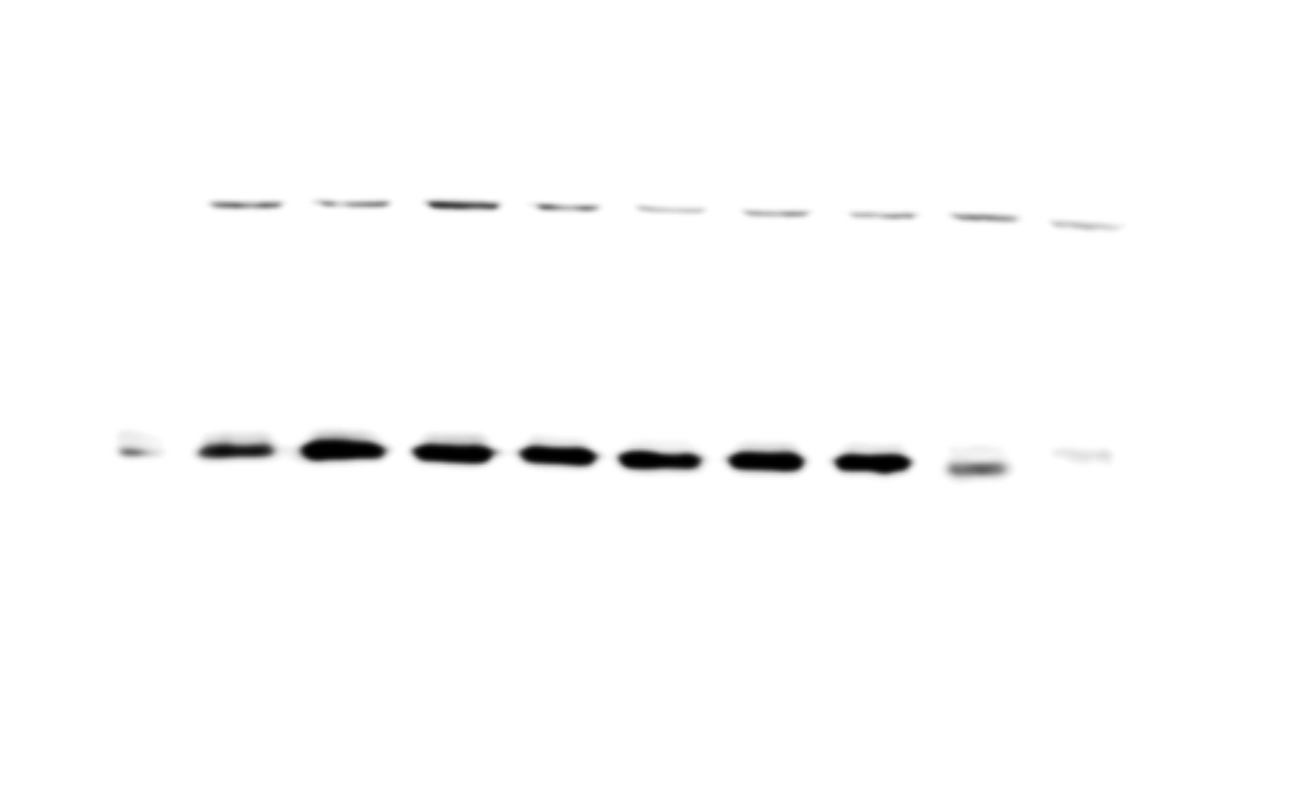

Supplement: Figure 7—source data 2. [file elife-99785-fig7-data2.zip › Figure 7-DHFR Line B Raw.tiff]

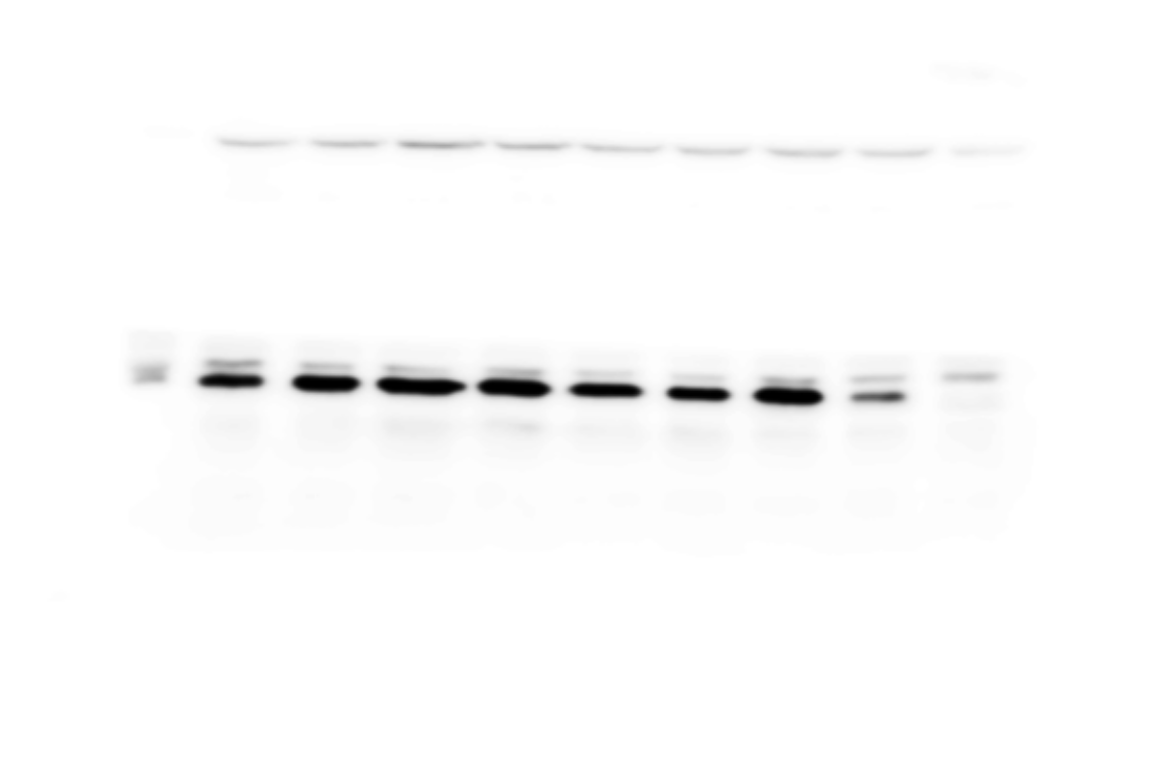

Supplement: Figure 7—source data 2. [file elife-99785-fig7-data2.zip › Figure 7-DHFR Line C Raw.tiff]

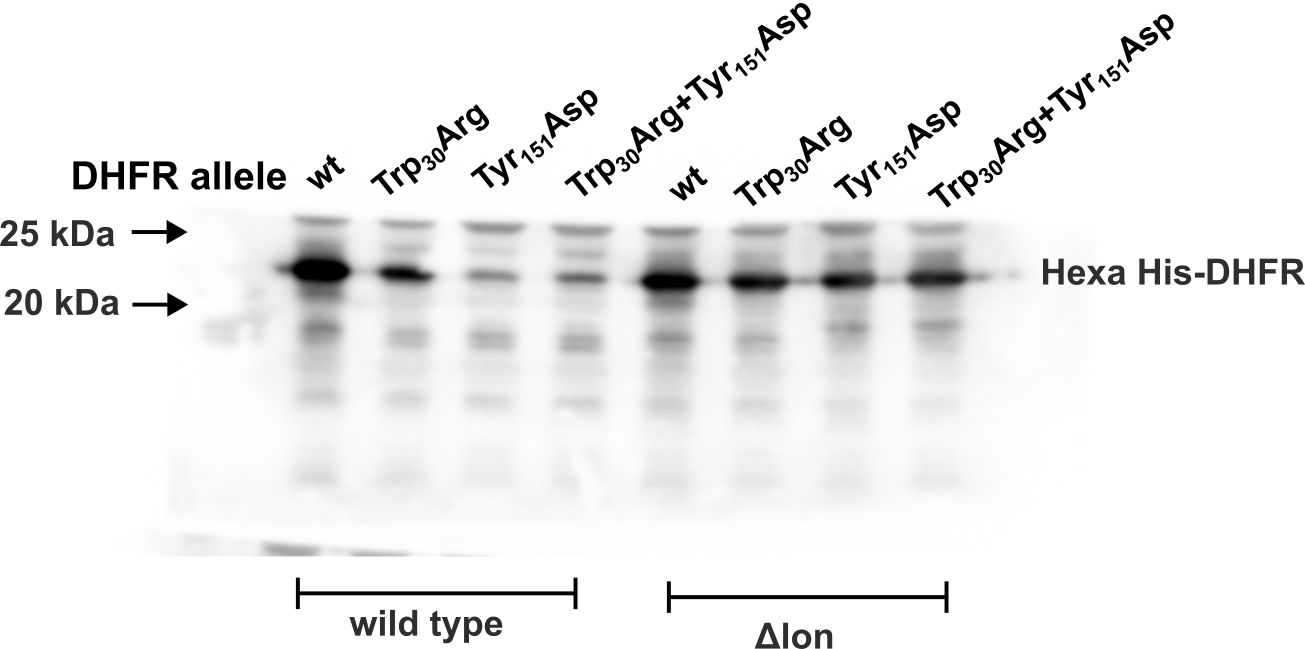

Supplement: Figure 8—source data 1. [file elife-99785-fig8-data1.zip › Figure 8-DHFR heterolous expression Annotated.tiff]

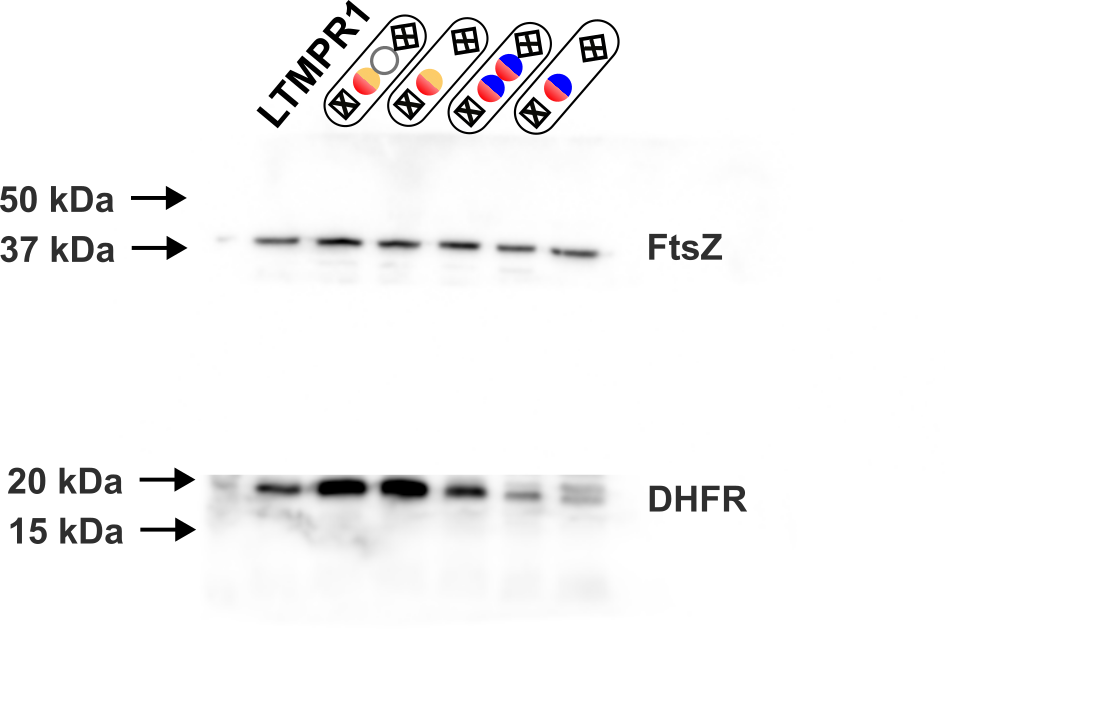

Supplement: Figure 8—source data 1. [file elife-99785-fig8-data1.zip › Figure 8-DHFR LTMPR1 derivatives Annotated.tiff]

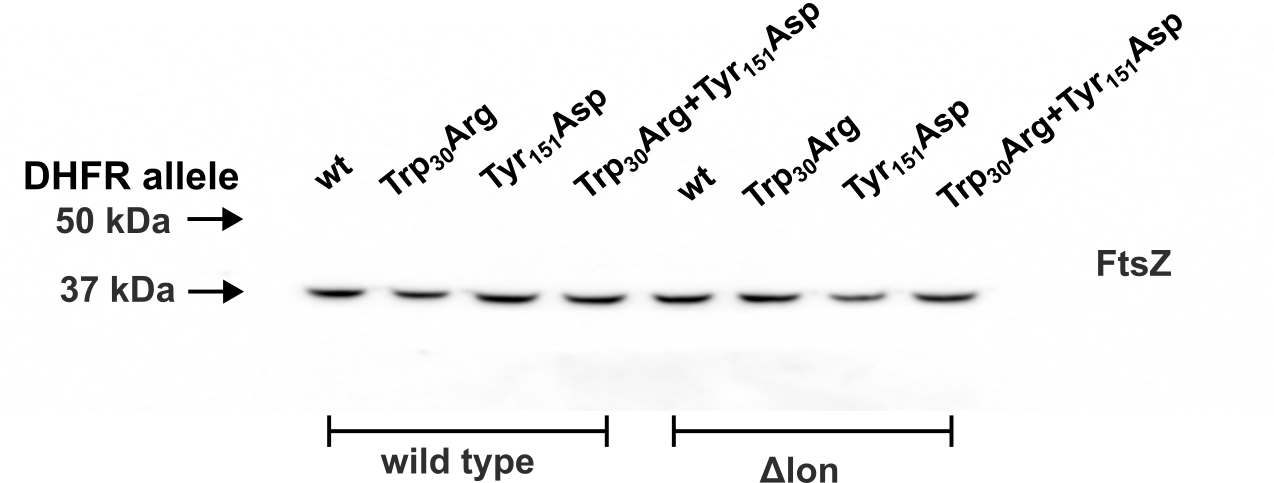

Supplement: Figure 8—source data 1. [file elife-99785-fig8-data1.zip › Figure 8-FtsZ heterolous expression Annotated.tiff]

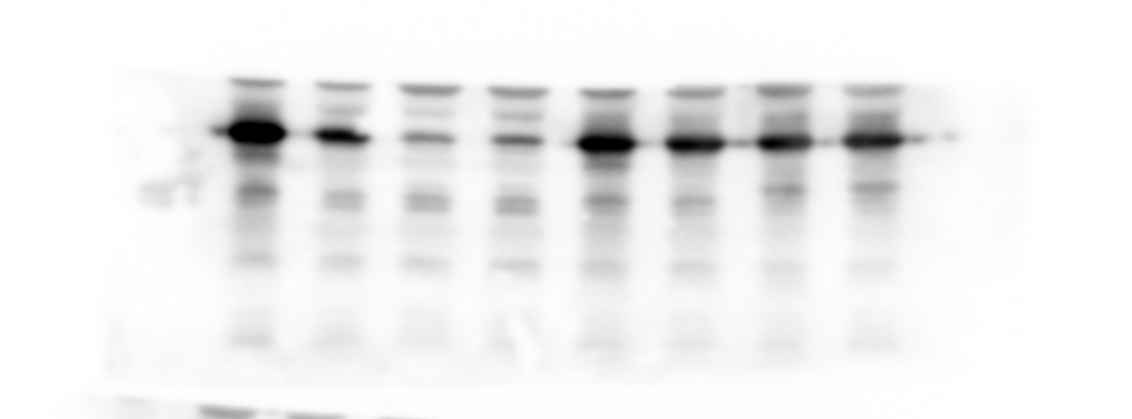

Supplement: Figure 8—source data 2. [file elife-99785-fig8-data2.zip › Figure 8-DHFR heterologous expression Raw.tiff]

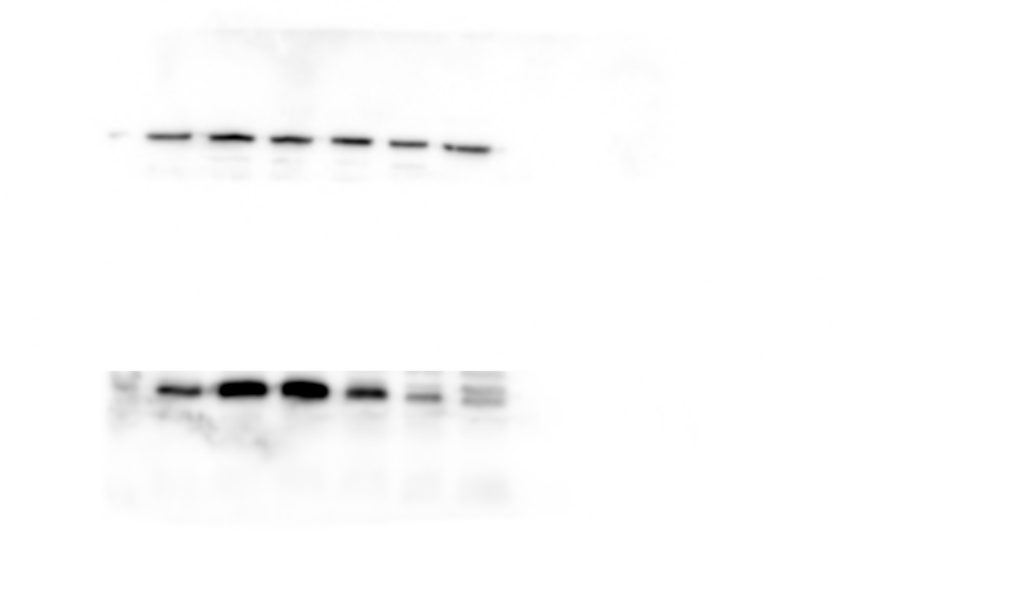

Supplement: Figure 8—source data 2. [file elife-99785-fig8-data2.zip › Figure 8-DHFR LTMPR1 derivates Raw.tiff]

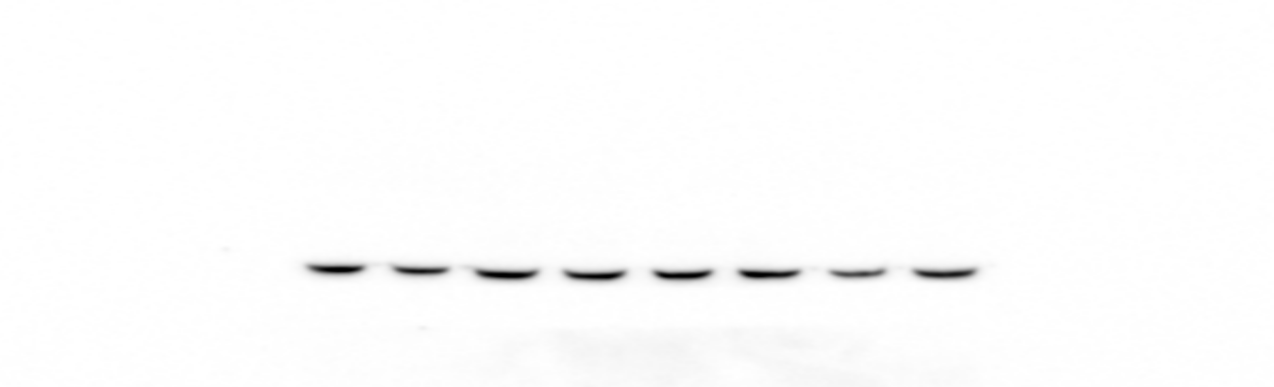

Supplement: Figure 8—source data 2. [file elife-99785-fig8-data2.zip › Figure 8-FtsZ heterologous expression Raw.tiff]
